# Laboratory capacity strengthening in Zimbabwe as part of the COVID-19 response: what has worked? What still needs to be done?

**DOI:** 10.11604/pamj.2022.43.85.35595

**Published:** 2022-10-18

**Authors:** Muchaneta Gudza-Mugabe, Mathias Dzobo, Agnes Juru, Lucia Sisya, Anderson Chimusoro, Raiva Simbi, Alex Gasasira

**Affiliations:** 1 World Health Organisation, Highlands, Harare, Zimbabwe,; 2 National Microbiology Reference Laboratory, Harare, Zimbabwe,; 3 Ministry of Health and Child Care, Harare, Zimbabwe

**Keywords:** COVID-19, Public health, Zimbabwe

## Abstract

The COVID-19 pandemic was declared a Public Health Emergency of International Concern on January 30, 2020. The government of Zimbabwe through the Ministry of Health and Child Care set up the COVID-19 national preparedness and response plan in which the laboratory was a key pillar. The implementation of PCR testing, genomic sequencing, and the establishment of quality management systems during the COVID-19 response strengthened the capacity of the public health laboratory system in responding to the pandemic. Here we present the different strategies taken by the government that strengthened laboratory capacity, the lessons learned during the COVID-19 response, and recommendations on how the capacity can be sustained and leveraged for outbreak response in the future.

## Commentary

COVID-19 disease, caused by the Severe Acute Respiratory Syndrome -Coronavirus-2 (SARS-CoV-2) was first reported in China in December 2019. On the 30^th^ of January 2022, World Health Organisation (WHO) declared the COVID-19 outbreak a Public Health Emergency of International Concern (PHEIC) under International Health Regulations (IHR) [1]. The Emergency Committee recommendations stated all countries should be prepared for active surveillance and early detection of COVID-19 [1]. The efforts to control COVID-19 in Zimbabwe were multisectoral and the laboratory was a key pillar; however, the process is still undocumented. Thus, this article discusses the strategies employed in handling the COVID-19 pandemic, and the strengthening of the public health laboratory system in Zimbabwe. The authors further discuss important challenges and propose recommendations to further strengthen the public health laboratory system beyond the current COVID-19 pandemic.

### Strategies to strengthen laboratory capacity during the COVID-19 pandemic in Zimbabwe

The development of the National COVID-19 Laboratory testing strategy and the review of the National Health Laboratory Strategy and policy documents in the context of COVID-19 played an important role in strengthening the laboratory in Zimbabwe. Tools for guidance and standard operating procedures for use during implementation were developed, reviewed, and approved as a matter of urgency, adopting WHO guidelines for SAR-CoV-2 infection detection.

Typical of health systems in sub-Saharan Africa, the capacity to respond to the COVID-19 pandemic in Zimbabwe was low due to inadequate funding and a lack of trained personnel to conduct COVID-19 testing and genomic sequencing [2]. Zimbabwe started testing for the SARS-CoV-2 virus in mid-February 2020 and the first case was reported on the 20^th^ of March 2020 [3]. The government of Zimbabwe, through the Ministry of Health and Child Care (MoHCC), set up the COVID-19 national preparedness and response plan that was composed of nine pillars. The laboratory pillar, led by the directorate of laboratory services (DLS) introduced a tiered system to decentralise COVID-19 testing by leveraging existing PCR testing platforms throughout the country. Overall, the national laboratory response to the COVID-19 pandemic was hinged on the introduction and decentralisation of antigen rapid diagnostic tests (Ag-RDT), participation in proficiency testing, acquisition, and repurposing of PCR platforms, and collaborative efforts with different organisations helped to strengthen the response to the pandemic.

### PCR Machines and Molecular Platforms/COVID-19 PCR testing

The Ebola virus disease (EVD) outbreak in West Africa provides useful lessons on how emergency responses can impact health systems, [4]. Novel technologies introduced to increase diagnostic capacity for EVD testing were leveraged in the COVID-19 response. In Zimbabwe, point-of-care platforms such as the GeneXpert (Cepheid) and other PCR platforms used in HIV viral load and Early Infant Diagnosis were leveraged to enable a rapid response to the pandemic. Before the beginning of the pandemic in Zimbabwe, there were a total of 10 Abbott m2000 RealTime Systems (Abbott), 6 Hologic Panther (Hologic), 6 BioMérieux (BioMérieux), and 145 GeneXpert machines. A further 10 Zhengkebio96 nucleic acid extractors (China), 10 Gentia (Gentia), 72 USTAR machines (EasyNAT), and 1 QuantStudio PCR (Thermofischer) platform were acquired to scale up COVID-19 PCR testing across the country. The implementation and scale-up of SARS-CoV-2 molecular testing were not without challenges as reagents stockout disrupted testing in some parts of the country due to supply chain inconsistencies caused by increased global demand for reagents and consumables. Given the existing capacity for molecular testing in the country, it will be important to sustain the use of the platforms to improve the overall diagnostic landscape in the country. Going forward it will be important to leverage the existing molecular testing platforms for diagnostic, teaching, and research purposes to improve the overall health system in Zimbabwe.

The Ebola virus disease (EVD) outbreak in West Africa provides useful lessons on how emergency responses can impact health systems, [4]. Novel technologies introduced to increase diagnostic capacity for EVD testing were leveraged in the COVID-19 response. In Zimbabwe, point-of-care platforms such as the GeneXpert (Cepheid) and other PCR platforms used in HIV viral load and Early Infant Diagnosis were leveraged to enable a rapid response to the pandemic. Before the beginning of the pandemic in Zimbabwe, there were a total of 10 Abbott m2000 RealTime Systems (Abbott), 6 Hologic Panther (Hologic), 6 BioMérieux (BioMérieux), and 145 GeneXpert machines. A further 10 Zhengkebio96 nucleic acid extractors (China), 10 Gentia (Gentia), 72 USTAR machines (EasyNAT), and 1 QuantStudio PCR (Thermofischer) platform were acquired to scale up COVID-19 PCR testing across the country. The implementation and scale-up of SARS-CoV-2 molecular testing were not without challenges as reagents stockout disrupted testing in some parts of the country due to supply chain inconsistencies caused by increased global demand for reagents and consumables. Given the existing capacity for molecular testing in the country, it will be important to sustain the use of the platforms to improve the overall diagnostic landscape in the country. Going forward it will be important to leverage the existing molecular testing platforms for diagnostic, teaching, and research purposes to improve the overall health system in Zimbabwe.

### Decentralised antigen Rapid diagnostic testing and reporting systems

The emergency use listing of two Ag-RDT, Abbott and SD Biosensor by WHO in September 2020 [5] led to the adoption of Ag-RDT for COVID-19 testing in Zimbabwe. The use of Ag-RDT enabled the decentralisation of COVID-19 testing to remote parts of the country thereby building capacity at the lower levels of the healthcare system. An initial 450 cadres from 38 facilities were trained in Ag-RDT testing and at the time of writing more than 6000 personnel from 1600 facilities around the country have been trained. The decentralisation of Ag-RDT testing enabled the establishment of a statistics collation system from the rural health centres and clinics up to the national level using social platforms like WhatsApp as an interim measure. These reporting systems may be used beyond COVID-19 in any other emergencies for disease surveillance and rapid outbreak response. However, with the limitation of network challenges that were faced, the offline database was established for reporting, which collects data from rural health centres and clinics up to the national level. Furthermore, the installation of a national GO-DATA system is ongoing in the country for both online and offline reporting. It is important to note that, the implementation of a program of this nature requires constant monitoring for instance at some sites testing was disrupted due to staff attrition and the repurposing of trained staff to attend to other hospital departments.

### Quality management systems set up and participation in proficiency testing

The COVID-19 response demanded strict adherence to quality when performing COVID-19 testing to ensure accuracy, reliability, and timely release of results. Before the COVID-19 pandemic in Zimbabwe, a few government and private laboratories were participating in external quality assessment programs (EQA) such as proficiency testing (PT). During the COVID-19 pandemic, the MoHCC and partners through the DLS facilitated the setting up of quality management systems (QMS) and participation of laboratories in PT programmes offered by organisations such as the WHO, Thistle (South Africa), and One World Accuracy (Canada). To standardise laboratory testing of COVID-19, Africa CDC in collaboration with ASLM and PanaBIOS as technical leads, introduced the COVID-19 Laboratory testing certification Program (CoLTeP) to ensure accuracy, and reliability of test results and that testing procedures be conducted under appropriate biosafety and biosecurity conditions [6]. A total of 34 laboratories were assessed under the CoLTeP programme and were certified to conduct COVID-19 testing for travelling individuals while other laboratories enrolled for the quality system assessment. Despite assessing a few components of the overall QMS, these programmes managed to promote the setup or revival of QMS, particularly in private laboratories. There is now an opportunity for continuous improvement of the QMS that was set.

### Partnerships and collaborations

The COVID-19 response brought about collaborations and partnerships in the form of inter-governmental, government to private, government to institutions and institution to institution collaborations. These partnerships strengthened the public health laboratory system in Zimbabwe in areas of the supply chain and logistics, training of laboratory personnel, sequencing, QMS, and outbreak response. Different partners came aboard to support the pandemic response by providing technical support for SARS-CoV-2 testing, outbreak response, building sequencing capacity, buying of reagents, and capacitating laboratory personnel e.g. the WHO, the Jack Ma Foundation [7], Biomedical Research and Training Institute (BRTI), and Clinton Health Access Initiative (CHAI). African Centre for Disease Control and Prevention (Africa-CDC), CDC, African Society for Laboratory Medicine (ASLM) (WHO, 2020, 2021; Africa CDC, 2021). The ASLM assessed COVID-19 testing facilities for adherence to biosafety and biosecurity requirements [8]. The Quadram Institute Biosciences (QIB) and Africa CDC supported SARS-CoV-2 sequencing efforts in the country [9]. The existing partnerships between the government and different organizations are necessary for sustaining a robust public health laboratory system. Beyond the current COVID-19 global pandemic. The government of Zimbabwe can bank on these important partnerships to address existing endemic diseases and respond to the next outbreak.

### Human resources

Early response to the COVID-19 pandemic in Zimbabwe was slowed due to a lack of skilled personnel to perform SARS-CoV-2 molecular testing among other reasons. Many laboratory cadres also lacked training and skills in disease outbreak response. Collaborative efforts with organisations such as the Kwazulu-Natal Research Innovation and Sequencing Platform (KRISP), QIB in the United Kingdom, and the Africa CDC facilitated the training of Zimbabwean scientists in genomic sequencing and bioinformatics analysis. It will be important to retain the trained staff through improving remuneration and working conditions and supporting continuous improvement towards building a resilient public health laboratory system capable of responding to future outbreaks.

### Recommendations

The COVID-19 response in Zimbabwe generated capacity and strengthened the public health laboratory system. However, the implementation of various strategies was not devoid of challenges. We propose recommendations to address some of the challenges and to maintain and improve existing capacity. Beyond the COVID-19 pandemic, a robust public health laboratory system will be important to address existing public health problems as well as respond to the next pandemic. 1)There is a need to maintain existing partnerships between the government and different local and international partners beyond the COVID-19 pandemic to enhance disease surveillance and cooperation in areas of public health interest. The government must maintain the existing partnerships with local private laboratories and research institutions to improve the overall surveillance and response efforts to disease outbreaks. The government through the DLS can play a role in supporting private laboratories to set up sustainable quality management systems with the goal of certification and accreditation. 2)The COVID-19 pandemic exposed the low capacity for genomic surveillance in Zimbabwe. There is an urgent need to invest in next-generation sequencing (NGS) platforms with high throughput and training of many laboratory cadres in performing sequencing and bioinformatics analysis of sequencing data [9]. Several training programs can be taken advantage of to train and build capacity in genomics such as the Human Heredity and Health in Africa (H3Africa) bioinformatics network training [9]. Genomic surveillance has the potential to detect circulating SARS-CoV-2 variants as well as to elucidate transmission dynamics [9]. Beyond COVID-19, sequencing is an important tool for confronting existing endemic infectious diseases such as HIV/AIDS and tuberculosis as well as understanding emerging global health threats such as antimicrobial resistance (AMR). 3)The COVID-19 response provided an opportunity for the training of cadres such as community healthcare workers in infection prevention control as well as Ag-RDT testing. This capacity at the lower levels of the health system is vital in ensuring service provision in areas that do not have trained and skilled laboratory cadres. However, there is a need for site support visits on an agreed time basis to assess competence and compliance with biosafety requirements when carrying out Ag-RDT testing and retrain cadres when necessary. It is also important to monitor testing sites to check if there is staff attrition which can hamper testing services. 4)Given the existing capacity in terms of PCR platforms and the potential for repurposing some of the machines for diagnosis of other infectious diseases or response to the next outbreak, the main challenge will be the availability of reagents and consumables. Beyond the COVID-19 pandemic, there is a need for a robust supply chain and inventory system as well as budgeting and forecasting mechanisms to prevent the shortage of essential reagents and other consumables. There is an urgent need for the country to generate capacity in Biotechnology and develop in vitro diagnostics such as rapid antigen kits at the innovation hubs set up in the country under the government´s drive to revive the economy through the provision of technical and research-based solutions.

### Conclusion

Before the COVID-19 pandemic, several deficiencies characterized the public health laboratory system in Zimbabwe. For instance, there were a few PCR and sequencing platforms as well as trained and skilled personnel in the areas of sequencing and bioinformatics analysis. However, during the pandemic, a significant shift in the paradigm of strengthening the public health laboratory system occurred characterised by improved laboratory infrastructure, training of laboratory cadres, and setting up of QMS and participation in EQA programmes, decentralised Ag-RDT testing, and important partnerships between the government and various organisations. In the post-pandemic period, further gains in strengthening the public health laboratory system can be achieved by constant capacity building in molecular techniques training, bioinformatics analysis, and setup maintaining of quality management systems. The current experiences have strengthened the public health laboratory system in Zimbabwe and this has laid the foundation for a significant post-COVID-19 transformation so that the public health laboratory system can be better prepared to address existing public health problems and the next global threat(s) of the 21^st^ century.
